# Real-time acidification monitoring through Sofar buoy and SAMI-pH integration

**DOI:** 10.1016/j.ohx.2026.e00772

**Published:** 2026-04-13

**Authors:** Taylor J. Gill, Mike Jankulak, John Osborne, Patrick M. Kiel, Ana M. Palacio-Castro, Ian C. Enochs

**Affiliations:** a University of Miami, Cooperative Institute for Marine and Atmospheric Studies, 4600 Rickenbacker Cswy., Miami, FL 33149, USA; b NOAA, Atlantic Oceanographic and Meteorological Laboratory, Ocean Chemistry and Ecosystem Division, 4301 Rickenbacker Cswy., Miami, FL 33149, USA; c University of Miami, Department of Marine Biology and Ecology, 4600 Rickenbacker Cswy., Miami, FL 33149, USA; d University of Washington, Cooperative Institute for Climate, Ocean and Ecosystem Studies (CICOES) under NOAA grant NA20OAR4320271, USA

**Keywords:** Monitoring, Ocean acidification, Restoration, Environmental change, Coral reefs

## Abstract

Ocean acidification (OA) impairs the ability of corals to build and maintain reef structures by reducing calcium carbonate deposition and accelerating the dissolution of existing frameworks. OA conditions can result from both natural pH fluctuations, driven by diel and seasonal variability in biological activity and water quality, and long-term increases in atmospheric CO_2_ absorption. Accurate characterization of OA requires precise, high-frequency time-series data, particularly in nearshore ecosystems where benthic community metabolism can cause rapid, localized shifts in carbonate chemistry. However, continuous, high-resolution pH monitoring remains challenging, and most existing technologies lack real-time feedback capabilities. Here, we present a real-time acidification monitoring system that integrates a Sofar Spotter buoy with a Sunburst SAMI-pH sensor. The system delivers continuous environmental data (benthic pH and temperature, surface temperature, wind, wave height, and barometric pressure) and sensor health diagnostics (battery levels and cellular connectivity status) to a public-facing dashboard. This system enables real-time access to high-frequency pH data and provides a modular and cost-effective alternative to larger, more complex platforms such as MAPCO_2_ buoys. Increased accessibility supports broader and more scalable monitoring efforts, supporting scientists, resource managers, and policymakers in tracking diel, seasonal, and long-term OA dynamics.

## Graphical abstract

**Figure:**
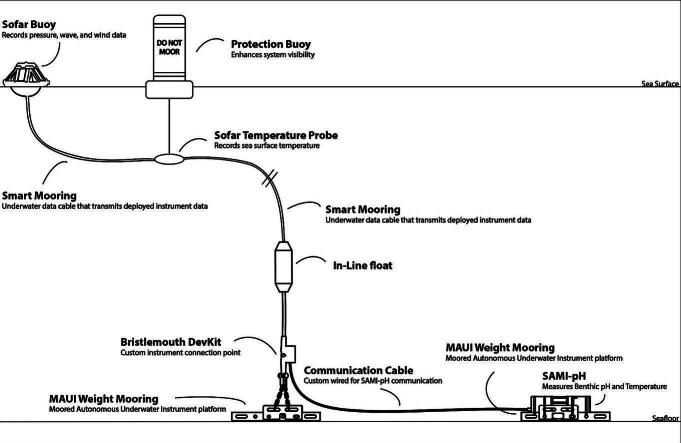

**Specifications table:** 

Hardware name	*ROAM System: Real-Time Ocean Acidification Monitoring System*
Subject area	Biological sciences (e.g., microbiology and biochemistry) Environmental, planetary and agricultural sciences Educational tools and open source alternatives to existing infrastructure
Hardware type	Field measurements and sensors
Closest commercial analog	*“No commercial analog is available.”*
Open source license	Creative Commons Attribution 4.0 International (CC BY 4.0)
Cost of hardware	*∼$32,000* *1. Buoy and mooring: ∼$14,000* *2. pH instrumentation: ∼$18,000*
Source file repository	https://doi.org/10.5281/zenodo.18838552

## 1 Hardware in context

While global ocean acidification is primarily driven by the uptake of anthropogenic CO_2_ [1], local dynamics, such as biological activity, restricted water exchange, and water quality, strongly influence carbonate chemistry at coastal scales [2]. These processes can reduce the ocean’s buffering capacity and accelerate changes in carbonate chemistry that threaten sensitive marine organisms, including corals and shellfish [3], [4]. In reef habitats, pH can strongly fluctuate over diel, seasonal, and interannual timescales, often reaching stress thresholds for marine life that surpass those of more stable open-ocean systems [5], [6], [7], [8]. High-resolution (minutes to hours) monitoring of local systems is essential for detecting long-term trends, capturing short-term variability, and identifying drivers of ecosystem change.

Detecting acidification trends across spatially complex reef systems requires monitoring strategies that capture both long-term patterns and high-frequency events. For example, an 11-year record from 38 sites across the Florida Coral Reef system has revealed substantial seasonal, interannual, and spatial variability, underscoring the value of sustained, distributed observations for informing local management [8]. However, discrete datasets alone cannot resolve high-frequency, periodic fluctuations (e.g., diel pH cycles, storm-driven fluctuations) that can strongly influence biological processes and ecosystem resilience [9]. These findings highlight the need for OA monitoring approaches that combine broad spatial coverage with the fine temporal resolution required to capture episodic events, capabilities that vary among existing methods, as outlined below.**

Current methods for monitoring ocean acidification include discrete sampling, autonomous sensors, and moored buoys, each with specific advantages and limitations. Discrete sampling methods, such as hand-collected bottle samples and CTD rosettes with Niskin bottles, offer high-accuracy measurements at targeted depths and time points [10]. However, these methods require a dedicated vessel, ranging from large research ships to smaller center-console boats, along with trained personnel and labor-intensive laboratory analysis using specialized, high-cost instruments, resulting in low temporal resolution and delayed data delivery. To address these limitations, programmable instruments like the Subsurface Automated Sampler (SAS) have been developed as an alternative to discrete sampling methods [11]. Although low-cost and autonomous, SAS units still rely on the same labor- and equipment-intensive laboratory processing as traditional bottle sampling and typically collect only two samples per deployment, increasing the risk of missing transient events. On the other end of the spectrum, moored platforms such as MAPCO_2_ buoys provide high-frequency, climate-quality surface data and valuable insight into short-term variability. However, their large size, high capital cost, substantial power requirements, and complex deployment and maintenance procedures, often require specialized vessels and technical crews that can limit their use in many locations. These platforms also rely on satellite telemetry, which increases operational expenses and can be cost-prohibitive for sustained, distributed monitoring [12].

Seawater pH has traditionally been measured using glass electrodes, which offer ±0.003 pH unit precision under laboratory conditions but degrade in marine settings due to drift and salinity effects, making them unsuitable for long-term, in situ monitoring [13]. Autonomous sensors based on ISFET technology (e.g., SeaFETs, Sea-Bird Scientific) can also provide high precision (∼±0.0005 pH [14]) but may exhibit long-term drift and require periodic recalibration, with additional maintenance and performance considerations described by Bresnahan et al. [15]. In contrast, the SAMI-pH (Sunburst Sensors, LLC [16]) is a colorimetric spectrophotometric sensor that provides slightly lower precision (±0.0007 to ±0.0014 pH) but long-term stability without recalibration, making it well-suited for extended autonomous deployments [17]. However, data from the SAMI-pH must typically be retrieved post-deployment, delaying access to critical information. Although the SAMI-pH can be integrated with MAPCO_2_ buoys for real-time transmission, such configurations inherit the high costs and logistical challenges of those moored platforms, including satellite telemetry expenses.

The integration of autonomous sensors into large-scale ocean observation networks is essential for expanding temporal coverage, enhancing monitoring efficiency, and ensuring the continuity of datasets critical for research and management [18], [19]. To bridge the gap between high-accuracy sensing and real-time data access, we integrated the SAMI-pH, a climate-quality spectrophotometric pH sensor that satisfies accuracy and stability requirements for detecting long-term trends, with the Sofar Spotter, a compact, solar-powered surface buoy equipped with cellular and satellite telemetry. This configuration enables hourly pH monitoring, captures high-frequency variability such as diel cycles and storm events, and supports sustained deployments in remote or logistically challenging environments. By pairing climate-quality measurements with real-time transmission in a lower-cost platform, the system addresses key gaps in ocean acidification monitoring and provides timely data to support science, management, and restoration efforts.

## 2 Hardware description

● Integrates the Sunburst SAMI-pH with the Sofar Spotter buoy to create a compact, low-cost, real-time pH monitoring system optimized for a variety of shallow water marine environments.
● Provides autonomous, hourly measurements of pH, benthic and surface temperature, wind speed, and atmospheric pressure via cellular telemetry.
● Uses a solar-powered Sofar Spotter buoy and an internally powered SAMI-pH, enabling long-term deployments in energy- and access-limited settings.
● Supports fast assembly, easy maintenance, and hardware customization through the modular design of both the Sofar Spotter platform and our custom integration. The Spotter’s architecture allows quick attachment of compatible hardware, while our design enables the custom cable to be adapted for other instruments and, with minor firmware changes, to operate a variety of additional sensors for site-specific adaptations.
● Delivers real-time data streaming, visualization, and diagnostics via a public-facing dashboard, enabling remote performance tracking and near-instantaneous ecological assessments to support time-sensitive decision-making.

**Fig. 1:**
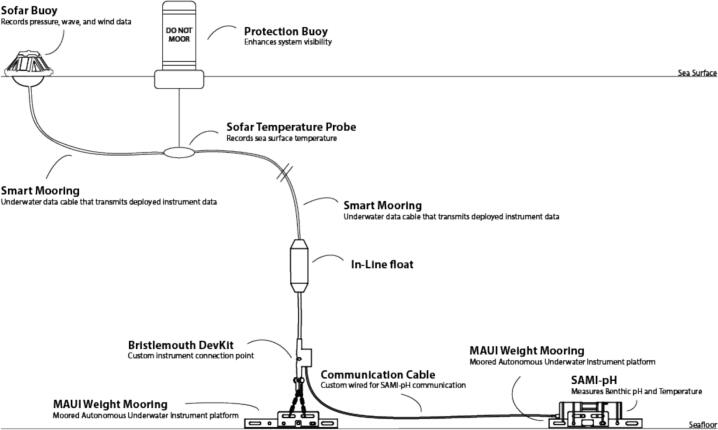
Fully integrated system diagram showing the Sofar Spotter buoy paired with the SAMI-pH sensor and Moored Autonomous Underwater Instrument (MAUI) weights.

The integrated OA monitoring system comprises a solar-powered Sofar Spotter buoy (Fig. 1) with a subsurface mooring line equipped with sensing hardware. The mooring can be configured with either two five m line segments or one five m line paired with a 10 m line segment **(Fig. 2.2 and 2.4), depending on site depth, and includes a Sofar temperature sensor **(Fig. 2.3) for collecting sea surface temperature data.

**Fig. 2:**
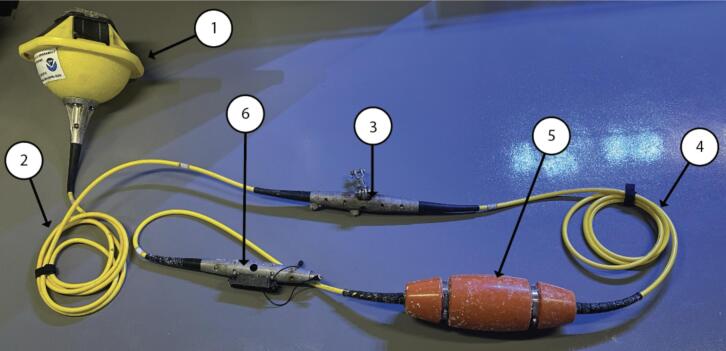
Assembly diagram of the Sofar Spotter buoy system, showing the primary components. The DevKit and in-line float are supplied separately. Numbered components include: (1) the Spotter buoy, (2) a five m smart mooring cable, (3) the center node housing the temperature probe, (4) a five m or 10 m smart mooring cable, (5) the in-line float, and (6) the bottom node.

**Fig. 3:**
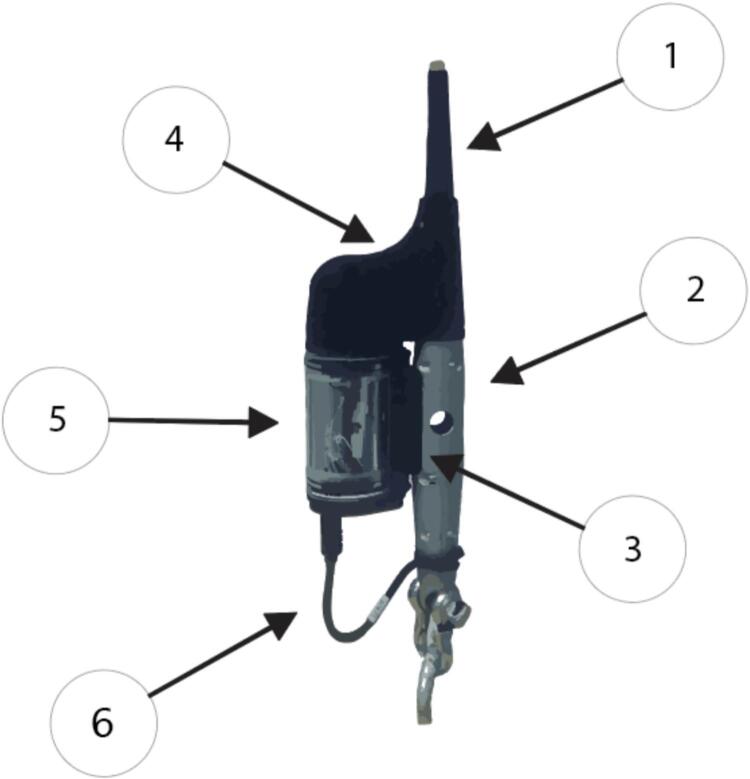
Annotated diagram of the DevKit. (1) Bottom end of the mooring line and data cable; (2) metal bottom node used to secure and support components; (3) mounting bracket for attaching the kit to the mooring infrastructure; (4) protective plastic cap/cover for the DevKit; (5) acrylic housing that contains and protects the circuit board; (6) custom communication cable connection point for Sofar/SAMI interface.

At the mooring’s base **(Fig. 2.6), a Bristlemouth-enabled Development Kit (DevKit) (Fig. 3) has been customized to interface with the Sunburst SAMI-pH sensor using RS-232 communication. “Bristlemouth-enabled” refers to the DevKit’s compatibility with the Bristlemouth open-ocean connectivity standard [20], which allows modular integration of sensors and devices through a standardized communication and power interface. A custom-fabricated cable (Fig. 3.6) connects the SAMI-pH to the DevKit, enabling automated data transfers to the Spotter buoy.

The buoy transmits this information via cellular and satellite telemetry to a public-facing dashboard. Designed with modularity and adaptability in mind, the system supports rapid deployment, minimal maintenance, and integration of additional sensors as needed. This system enables real-time insights into environmental data and system diagnostics, facilitating more reliable autonomous deployments.

### 2.1 System components

#### 2.1.1 Sofar Spotter buoy and Bristlemouth Development Kit

The Spotter buoy (Sofar Ocean [21]) is a lightweight (7.45 kg) remote sensing platform designed to collect real-time ocean data, including wave spectra (ft), wind speed (mph), barometric pressure (hPa), sea surface temperature (°F), and GPS location. The buoy is constructed from UV-resistant plastics with corrosion-resistant components. It is powered by four solar panels connected to a rechargeable lithium-ion battery, enabling multi-month to year-long deployments. Operational longevity depends on environmental conditions; however, deployments of six-twelve months require routine inspection and maintenance. Data transmission is supported via cellular or satellite networks, depending on the deployment site.

The buoy is equipped with Bristlemouth, an open-standard hardware and software interface developed to support modular, plug-and-play interoperability across ocean sensors, power modules, and communication systems. Bristlemouth is integrated directly into the Spotter’s hardware and firmware, allowing external devices to connect via standardized jumper cables that provide secure, corrosion-resistant electrical and data connections. This modular design enables users to easily swap or add components, such as Bristlemouth-enabled sensors or power modules, without requiring custom wiring or reprogramming, making it simple to customize the buoy for specific mission requirements.

A Bristlemouth-enabled Development Kit (DevKit) (Fig. 3) was used in this deployment to connect the SAMI-pH sensor. The DevKit features Bristlemouth-compatible ports that allow it to interface with the buoy via jumper cables. It features a custom circuit board housed in a waterproof enclosure and communicates with the sensor using the RS-232 protocol. This configuration enables reliable real-time data transmission from instruments through the Spotter’s telemetry system.

#### 2.1.2 Sunburst SAMI-pH sensor

The SAMI-pH sensor (Sunburst Sensors, LLC [16]) is a compact, autonomous instrument that measures seawater pH and temperature using a colorimetric indicator dye method. Each unit is factory-calibrated and, in this deployment, was programmed in-house to collect measurements at 60-minute intervals. The sensor includes a copper intake filter to minimize biofouling and prevent debris from entering the fluid path, helping to maintain calibration and data quality over extended deployments.

#### 2.1.3 Mooring configuration

The system is moored using either stainless steel pins or MAUI (Moored Autonomous Underwater Instrument) weight systems, originally developed and deployed in support of the NOAA Coral Reef Ecosystem Integrated Observing System in the Pacific (Fig. 1). The choice of mooring method depends on site-specific conditions such as depth, substrate type, wave exposure, and the need to avoid contact with the reef. One configuration had the SAMI-pH mounted to a fixed MAUI weight system with the buoy attached to a single stainless steel mooring pin fitted with a D-shackle. This configuration was selected over a swivel connection due to the large and variable wave heights and storm surges at the deployment sites, which could cause excessive rotation or torsion in a suspended system. In addition, the SAMI-pH’s size and weight, combined with the importance of maintaining a fixed sampling location, made mounting it directly on the mooring line impractical. The fixed mount keeps the instrument stable on the seafloor, ensuring consistent sampling height and reducing motion-induced variability in measurements. The D-shackle also prevents the communication cable from twisting around the mooring, thereby avoiding mechanical stress or abrasion damage to both the instrument and cable. The Sofar Spotter buoy was secured separately with a stainless steel mooring pin, positioned approximately five m from the reef and two m from the SAMI-pH to reduce collision risk during high wave conditions. A custom three m communication cable connected the SAMI-pH to the buoy, providing sufficient slack to prevent strain while keeping the cable clear of entanglement hazards.

The Spotter buoy system supports a range of mooring configurations to accommodate different site conditions. Downlines can extend up to 130 m using combinations of five m to 65 m line segments. When using a Bristlemouth-enabled DevKit, the maximum supported working depth is 75 m due to pressure limitations. For shallow reef deployments (∼six m), we used two five m line segments, which also helped reduce the spacing between the buoy and its associated surface components.

In all deployments, an optional surface protection float was positioned adjacent to the Spotter buoy to reduce the risk of vessel strikes, entanglement, and wave-induced damage. In high-traffic areas, tall Taylor Made Sur-Mark marker buoys with lights were used to maximize visibility to boaters. This configuration significantly improved surface visibility and reduced collision incidence without compromising sensor function or data quality (see Fig. 1 for placement).

#### 2.1.4 Wiring and integration

The bottom cap of the DevKit was drilled using a 25/64-inch bit and tapped to create threads that accommodate a six-pin female bulkhead connector, ensuring waterproof communication between the Spotter buoy and the SAMI-pH sensor. Internal wiring provided ground and RS-232 communication lines (Tx/Rx) between the DevKit and the SAMI-pH. All connections were tested for continuity and signal integrity before deployment.

#### 2.1.5 Power and sampling schedule

While the SAMI-pH sensor runs on its internal battery, the DevKit receives power from the Spotter buoy via a Smart Mooring Cable. This protected line enables both data and power transmission between the surface and subsurface components. To conserve energy, the buoy’s internal power controller, which manages electrical delivery to Bristlemouth-connected devices, was configured to activate once per hour in alignment with the SAMI-pH’s 60-minute sampling schedule. During each activation, the controller powers the DevKit long enough to allow data transmission from the sensor, then shuts off until the next scheduled interval. This approach ensures that the sensor and buoy operate in sync while minimizing unnecessary power usage.

### 2.2 Software

The software system developed for this project enables fully automated data collection, transmission, processing, and visualization of seawater pH in near real-time. Data flow begins with the SAMI-pH sensor collecting hourly measurements, which are transmitted through the Spotter buoy using custom firmware on the Bristlemouth-enabled DevKit. Once transmitted via cellular or satellite telemetry, the data are retrieved from the Sofar Ocean API, decoded, and stored in a centralized database. These processed data are then visualized through a public-facing dashboard, allowing researchers and stakeholders to monitor environmental conditions at reef sites in near real-time.

#### 2.2.1 DevKit firmware (BMDK to Spotter)

A critical component of the integration between the SAMI-pH sensor and the Spotter buoy is the custom firmware running on the Bristlemouth-enabled Development Kit (DevKit). Written in C++, the firmware was developed using guidance and example code structures from Bristlemouth’s tutorial documentation. The custom firmware initializes a serial connection with the SAMI-pH and remains in a listening state, awaiting data output from the sensor.

The hourly SAMI-pH measurements are collected during a sampling cycle of approximately two minutes. During this time, the sensor transmits two messages: a short status message indicating the start of measurement and a longer data message containing the complete set of output values. The DevKit firmware receives this serial input one character at a time, storing it in a buffer. A transmission is triggered when either an end-of-line character is detected (newline (ASCII 10) or a carriage return (ASCII 13)) or the buffer reaches 295 bytes, a safety limit chosen based on the DevKit’s 311-byte maximum transmission size.

When a transmission is triggered, the firmware routes the buffered message to three destinations: the DevKit console (used for debugging), the Spotter buoy console, and a local data file on the buoy’s microSD card. In parallel, it initiates data transmission via the Spotter’s built-in telemetry system, which primarily uses cellular networks but automatically switches to Iridium satellite as a fallback when cellular service is unavailable. In addition to these near real-time outputs, the SAMI-pH also logs all data internally, providing a secondary record for post-deployment validation and verification.

The firmware is compiled following the instructions provided in the Bristlemouth developer documentation [22]. Once compiled, the resulting binary is loaded onto the Spotter’s SD card and flashed to the DevKit’s onboard microcontroller via a serial connection using the Bristlemouth command-line interface (CLI) during pre-deployment configuration.

#### 2.2.2 Data acquisition (API to database)

After data are transmitted from the buoy via cellular or satellite telemetry, they are stored on Sofar Ocean’s cloud platform and can be accessed programmatically through their publicly documented API (https://api.sofarocean.com/api/). A data acquisition python script, provided in the supplemental materials, automates this process. Given a Spotter ID and API token, the script queries the Sofar API for a specified time range and downloads all associated buoy data, including wind speed, wave height, barometric pressure, surface temperature, and pH measurements from the integrated SAMI-pH sensor.

The SAMI-pH transmits its data as a hexadecimal string. Its exact format varies by firmware version and controller board revision. The previously mentioned data acquisition script is currently configured to decode the 466-byte format used by recent SAMI-pH units, though it can be modified to accommodate older models. The decoded data stream includes a timestamp, two temperature values (measured before and after reagent mixing), salinity, battery voltage, and a series of signal intensities and reference values at 434 nm and 578 nm wavelengths. The first four spectral measurements are “blank” readings of ambient seawater; the remaining measurements are collected after the sample has been mixed with a colorimetric indicator reagent. A pH value is then calculated using the absorbance ratio method based on algorithms and logic provided by Sunburst Sensors.

Once decoded, the resulting data, which includes metadata, environmental parameters, and pH measurements, are uploaded to a MySQL relational database hosted on a local server. This database supports both near real-time and historical data access for visualization and analysis. New pH readings typically become available within five minutes of collection. They are streamed to a dashboard, where they can be viewed interactively by scientists, managers, and the public. Data from four systems deployed as part of the Mission: Iconic Reefs Environmental Monitoring program can be viewed at https://coral.aoml.noaa.gov/mir/.

It is important to note that pH values displayed on this dashboard represent preliminary data derived from raw data before quality controls are applied. These values are suitable for real-time trend monitoring but may not meet the standards of research-grade quality. For high-accuracy scientific use, data logged internally by the SAMI-pH are downloaded post-deployment and processed using Sunburst Sensors’ QC_PH software [16], which applies manufacturer-recommended quality control protocols, blank corrections, calibration adjustments, and uncertainty estimates.

#### 2.2.3 Data visualization (database to public-facing Shiny application)

**Fig. 4:**
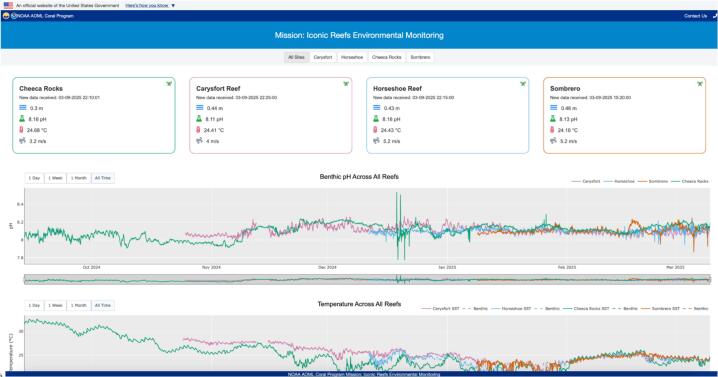
Public dashboard displaying data from each of the Mission: Iconic Reef Environmental Monitoring buoys. Accessible at: https://coral.aoml.noaa.gov/mir/. Data depicted in this figure are preliminary real-time measurements and have not undergone full post-deployment calibration or quality control.

The data collected by the buoys are visualized in near real-time through an interactive web dashboard (Fig. 4) built in RStudio (version 2024.04.0) [23] using the Shiny framework (version 1.10.0) [24] and Plotly [25] for dynamic graphics. The application retrieves data directly from a MySQL database, updating automatically as new values are processed, typically within a few minutes of each measurement.

**Fig. 5:**
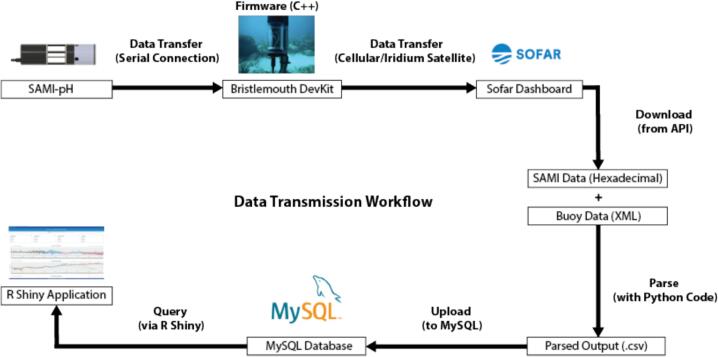
Transmission workflow from instrumentation to public dashboard, illustrating real-time data flow from sensor deployment through parsing and database integration.

The dashboard includes a multi-reef overview page that displays key environmental parameters (e.g., pH, sea temperature, wave height, wind speed, and barometric pressure) across all monitored sites. Individual reef-specific pages allow users to explore data for a single location in more detail, access contextual information about each reef, and examine trends in isolation. All plots are interactive via Plotly, enabling users to zoom, pan, and highlight specific time frames of interest. A data flow diagram is provided in Fig. 5.

## 3 Design files summary

All design files associated with this monitoring system are listed in Table 1 and described in detail below.

**Table 1:** Design files.

**Design filename**	**File type**	**Open source license**	**Location of the file**
*Parsing Code*	*.py*	CC BY 4.0	https://doi.org/10.5281/zenodo.18838552
*C++ User Code*	*.cpp*	Apache 2.0	https://doi.org/10.5281/zenodo.18838552
MAUI Weight Design	*.step*	CC BY 4.0	https://doi.org/10.5281/zenodo.18838552
Database Code	*.sql*	CC BY 4.0	https://doi.org/10.5281/zenodo.18838552
Visualization Code	*.R*	CC BY 4.0	https://doi.org/10.5281/zenodo.18838552

*The* ***Parsing Code,*** *written in python, is used to connect to the Sofar API, download the data, and process the raw SAMI-pH strings into values.*

*The* ***User Code****, written in C++, is compiled as part of the firmware and defines the custom behaviors of the DevKit. It controls how the device interacts with sensors, processes data, and communicates over the Bristlemouth network.*

*The* ***MAUI Weight Design*** *is a CAD file that illustrates the construction of this mooring system. The starboard mounts were cut in-house using a CNC machine, and the lead weights were custom molded through a local welding company.*

*The* ***Database Code*** *defines seven relational tables, for a MySQL database, that can be used to organize data from the Sofar API and the site and timing metadata associated with each deployment.*

*The* ***Visualization Code*** *is written in R/Shiny and provides an interactive dashboard template for visualizing SAMI-pH and Spotter buoy time-series data.*

## 4 Bill of materials summary

The system supports two deployment configurations: one stainless steel mooring pin with one MAUI Mooring set (the setup used in our deployments) or two MAUI Mooring weight sets without a pin. The components for this system are listed in Table 2.

**Table 2:** Bill of materials.

**Designator**	**Component**	**Number**	**Cost per unit – USD**	**Total cost – USD**	**Source of materials**	**Material type**
	Sofar Spotter Buoy	1	$6,600	$6,600	Sofar Oceans	Other
	DevKit	1	$1,995	$1,995	Sofar Oceans	Other
	Temperature Node	1	$795-$845	$795-$845	Sofar Oceans	Other
	5 m Mooring Line	1	$550	$550	Sofar Oceans	Other
	10 m Mooring Line	1	$650	$650	Sofar Oceans	Other
	In-Line Float	1	$795	$795	Sofar Oceans	Other
	Surface/Protection Float	1	N/A	N/A	Optional Addition	Other
	SAMI-pH Sensor	1	$18,000	$18,000	Sunburst Sensors	Other
	Communication Cable with Bulkhead	1	$1,129	$1,129	Teledyne	Belden
	MAUI Mooring Weights	2	$649	$1,299	Local Welding Company	Lead
	MAUI Mooring Side Mounts	2	$68.85	$68.85	USA Plastics	Starboard
	MAUI Mooring Connectors: 1 bolt, 1 locking nut, 1 hex nut, 2 washers per point	4	$3.02	$14.96	Grainger	316 T Stainless Steel
	Mooring Pin	1	$6	$6	Local Welding Company	316 T Stainless Steel

No formal designators are used in the associated design files; components are listed by name.

## 5 Build instructions

This manuscript provides a high-level overview of the system architecture and integration workflow and is not intended to serve as fully standalone assembly documentation. Detailed build instructions for the monitoring system with complete step-by-step procedures are provided as a PDF document (ROAMBuildManual.pdf) in the source file repository.

## 6 Operation instructions

Safety: Personnel should be aware of the following hazards: (1) electrical hazards associated with operating an electronic system in a marine environment; (2) chemical exposure during methanol handling for filter flushing; (3) irritation from cement mixtures; (4) use of underwater power tools; and (5) marine environment hazards during SCUBA deployment. Appropriate PPE and safety protocols should be followed.

### 6.1 System testing

Before deployment, the SAMI-pH was calibrated during a two-day in-house procedure in a recirculating seawater bath. During the first 24 h, the sensor acclimates to its surroundings. On the second day, bottle samples are collected every two hours and analyzed for pH and salinity using spectrophotometry (Agilent Technologies Cary 8454 UV–Vis) and densitometry (Anton Paar DMA 5000 M), following the standardized procedures outlined in Dickson et al. [10].

Additional cross-validation was conducted using a YSI EcoSense EC300A probe (temperature and salinity) and SBE 56 temperature loggers (Sea-Bird Scientific) deployed in the same bath alongside the SAMI-pH. Following data collection, SAMI-pH pH measurements were compared to laboratory spectrophotometric pH, SAMI-pH temperature readings were compared to both the YSI probe and SBE 56 sensors, and salinity from the water samples was compared to the YSI probe measurements. These comparisons were used to verify instrument agreement, confirm calibration accuracy, and detect any measurement drift prior to field deployment.

Following calibration, the fully assembled system was tested for energy consumption and data transmission over a 24-hour period. The buoy was placed in full view of the sun with unobstructed sky access to ensure solar charging. The SAMI-pH was immersed in a saltwater bath, and data transmission, power cycling, and battery recharge status were monitored through the Spotter dashboard. This test confirmed that the system could maintain its sampling schedule without energy deficits.

### 6.2 SAMI priming and flushing

On the day before deployment, the SAMI-pH is connected to a computer via the SAMI Client Software to check for airlocks or tubing obstructions. Under the “Utility” tab, the cycle count is set to 10, and deionized (DI) water is pushed through the system via a syringe at constant pressure. If the flow is confirmed, the cycle count is increased to 99, and the instrument is flushed with DI water or pre-filled bags. Immediately before deployment, the intake filter is flushed with three rinses of methanol, followed by three rinses of deionized (DI) water, to ensure proper wetting. This step is critical for accurate post-deployment sampling, as initial measurements are often affected by residual air or dry filters. The longevity of the copper intake filter depends on the deployment environment and experimental design (e.g., sampling frequency and deployment duration). In tropical reef environments with hourly sampling, the intake filter has typically remained effective for six to nine months, as indicated by post-deployment inspections. However, inspection is recommended whenever the device is serviced or recovered from the water.

### 6.3 Mooring deployment

Depending on environmental conditions, either a stainless steel mooring pin or MAUI weight system is used. Mooring pins are selected for sites with stable, exposed hard-bottom substrate that allow secure installation close to the reef without risk of damaging coral. MAUI weights are used in sandy environments where there is no suitable hard-bottom location to install a pin close enough to the reef while still avoiding harm to living coral.

When using a mooring pin, a NEMO Hammer Drill is used to make a hole in the substrate. The stainless steel pin is then inserted and secured with a one-to-one mix of Type I/II Portland cement and seawater prepared topside and transported underwater in 16-inch piping bags. Divers squeeze the cement mixture directly into the drilled hole around the pin until it is fully encapsulated, ensuring minimal void space. The cement is allowed to cure in situ for at least 48 h prior to instrument connection. If MAUI weights are used, divers pre-stage the system underwater and ensure the mooring site is secure before connecting the instruments.

### 6.4 System connection and assembly

All instrumentation is connected in the air before deployment. Two divers are required: one to manage the SAMI-pH and one to position the DevKit and buoy. Care is taken to avoid pulling the SAMI filter off. The buoy and associated mooring line are lowered into the water, and the underwater team positions each component at the deployment site.

### 6.5 Final deployment

The DevKit and buoy bottom node are attached to the anchor point using a half inch stainless steel D-shackle, secured with a zip tie to prevent loosening. The SAMI-pH is mounted to the MAUI weights using stainless steel hose clamps passed through side-mount brackets, supplemented by zip ties for redundancy and vibration dampening. Cable slack is carefully managed and zip-tied to both anchor points to minimize strain on connectors and prevent overextension during large swell events.

### 6.6 Post-deployment validation

Upon deployment, discrete water samples are collected adjacent to the SAMI-pH sensor at the time of scheduled readings (e.g., precisely on the hour) to support post-deployment data validation. These samples are analyzed in the laboratory, where pH is measured spectrophotometrically and salinity is determined through densitometry (see system testing section). Laboratory pH results are then paired with the known site and timestamp of collection and compared directly against the corresponding SAMI-pH readings. All bottle data are archived in the long-term database for reference and quality assurance.

## 7 Validation and characterization

**Fig. 6:**
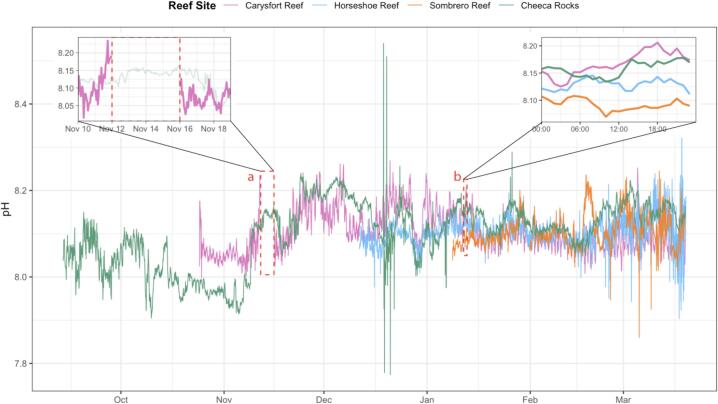
Benthic pH measurements at four reef sites over time. Inset a represents an instance where there was a data gap following a boat collision with the Carysfort Reef buoy. Inset b shows the benthic pH measurements at four reef sites over time shown over one day. These data are preliminary real-time measurements and have not undergone full post-deployment calibration or quality control.

Four fully integrated buoy systems have been successfully deployed at reef sites spanning the upper, middle, and lower Florida Keys between September 2024, and January 2025 as part of NOAA’s Mission: Iconic Reefs initiative. The first system was installed at Cheeca Rocks (2024-09-13), followed by Carysfort Reef (2024-10-24), Horseshoe Reef (2024-12-11), and Sombrero Reef (2025-01-08). Each system operates autonomously, sampling and transmitting seawater pH, temperature, wave height, wind speed, and barometric pressure, on an hourly basis. Fig. 6 shows diel pH variability across the four reefs during a representative 24-hour period in the upper right inset.

One specific use case in which this integration has enhanced environmental monitoring is by enabling timely replacement of SAMI-pH sensors based on real-time battery level readings, ensuring continuous data collection and reducing downtime. Traditional instrument deployment methods do not provide real-time access to battery and diagnostic information for these instruments, which could lead to sensors being retrieved too early or too late, resulting in valuable data being missed, or to non-functioning sensors left in the water, failing to collect any data. With this level of operational insight, battery levels from the SAMI-pH are logged hourly and available in real time, allowing field teams to schedule sensor swaps during optimal windows, reducing data loss and maximizing deployment time.

The system’s performance was characterized across multiple operational parameters. The SAMI-pH sensor and Sofar Spotter buoy are commercially validated instruments; therefore, the validation presented here focuses specifically on the performance of the integration components developed in this work, including firmware communication, modified DevKit hardware interfaces, and the data ingestion pipeline. Rather than re-evaluating the intrinsic accuracy of the commercial instruments, we assess the robustness of the communication link, system uptime, survivability of hardware modifications, and operational reliability under field conditions. For instance, data transmission reliability was assessed under varying weather conditions and sea states by monitoring incoming data streams through the system’s dashboard, with particular attention during known storm events or periods of high swell. Design adaptations were implemented based on site-specific challenges; for example, five recorded boat strikes across three sites prompted testing of alternative buoy configurations with increased surface visibility and reinforced impact resistance. This included adding large Taylor Made Sur-Mark marker buoys equipped within a flashing light for greater visibility and securing them to the Sofar mooring line with Kevlar line for enhanced durability. These changes improved survivability in high-traffic areas without compromising data integrity.

To quantitatively assess communication reliability, transmission performance was evaluated during the first three months following deployment at each site. Across the four buoy systems, 63–99% of scheduled hourly pH measurements were successfully transmitted in near real time (63%, 69%, 92%, and 99%). When including post-recovery uploads from onboard storage, total data coverage ranged from 91 to 99% of expected hourly measurements (91%, 95%, 98%, and 99%). Periods of reduced real-time transmission corresponded to documented field interruptions (e.g., communication cable damage, cellular service interruptions, or vessel impacts) rather than failures of the firmware integration or hardware modifications introduced in this work.

### 7.1 Hardware capabilities

● Sampling interval: The SAMI-pH and Spotter buoy were configured for synchronized one-hour logging. The SAMI-pH supports programmable intervals ranging from every 15 min to once per day (24 h), allowing customization based on deployment duration and power availability. The Spotter buoy supports continuous telemetry or customizable transmission schedules.
● Deployment longevity: Approximately nine months per SAMI-pH battery cycle (dependent on sampling frequency and site conditions).
● Power system: Solar-powered Spotter buoy with real-time voltage reporting and battery diagnostics. The SAMI-pH operates independently with its own internal battery system (12 V).
● Telemetry: Cellular; real-time API integration with less than five-minute delay under normal conditions.
● Sensor integration: RS-232 communication via custom DevKit wiring; additional sensor integration possible with minor firmware/software updates.
● Deployment depth: Tested at ∼six m depth; DevKit pressure tested to 2.5 bar (∼25 m)
● pH Accuracy and Precision: ±0.003 pH units accuracy (based on CRM intercomparison; precision typically <0.001 pH units over six months (based on manufacturer specs).

### 7.2 Hardware limitations

Despite the system’s robust performance, several limitations and challenges remain:

• Subsurface failure diagnosis- While integrated telemetry provides real-time battery voltage, sampling status, and communication logs, certain failures can only be confirmed through on-site inspection. For example, a sudden loss of communication from a SAMI-pH sensor (Fig. 6, upper left inset) prompted a site visit, where the team discovered a severed mooring line and abrasion damage to the communication cable, likely caused by a vessel strike.
• Telemetry outages during storms- Cellular telemetry is vulnerable during certain storm events, leading to temporary data transmission gaps. To mitigate this, the system includes an Iridium satellite fallback that maintains transmissions when cellular networks are unavailable. Cellular service can often be restored remotely, but in some cases requires manual intervention.
• Environmental durability- High boating activity and biological fouling, such as barnacle growth on sensors, can degrade system materials and necessitate mid-deployment maintenance. Prolonged seawater exposure also poses corrosion risks to metal components and can reduce the efficiency of solar panels.
• Bandwidth limitations- Integrating multiple high-frequency sensors can exceed both cellular and satellite transmission capacity, requiring the system to send fewer data points or group data into larger, less frequent transmissions to ensure successful delivery.

## Funding

This research was supported by funding from the Coral Reef Conservation Program (CRCP, grant #31520 to I.C. Enochs), which provided essential resources for the development and deployment of the monitoring system. This research was carried out in part under the auspices of the Cooperative Institute for Marine and Atmospheric Studies, a cooperative institute of the University of Miami and the National Oceanic and Atmospheric Administration (NOAA), cooperative agreement NA 20OAR4320472.

## Declaration of competing interest

The authors declare that they have no known competing financial interests or personal relationships that could have appeared to influence the work reported in this paper.
