# Supplementary material for: Real-time acidification monitoring through Sofar buoy and SAMI-pH integration
Source: HardwareX. 2026 Apr 13;26:e00772. doi: 10.1016/j.ohx.2026.e00772 (PMC13101671; doi:10.1016/j.ohx.2026.e00772)
Supplement: Supplementary Data 1 [file mmc1.pdf]

Real-Time Ocean Acidification Monitoring System: Sofar Buoy and SAMI-pH Integration

Build & Coding Instructions

Last updated by TJG as of 03/05/26

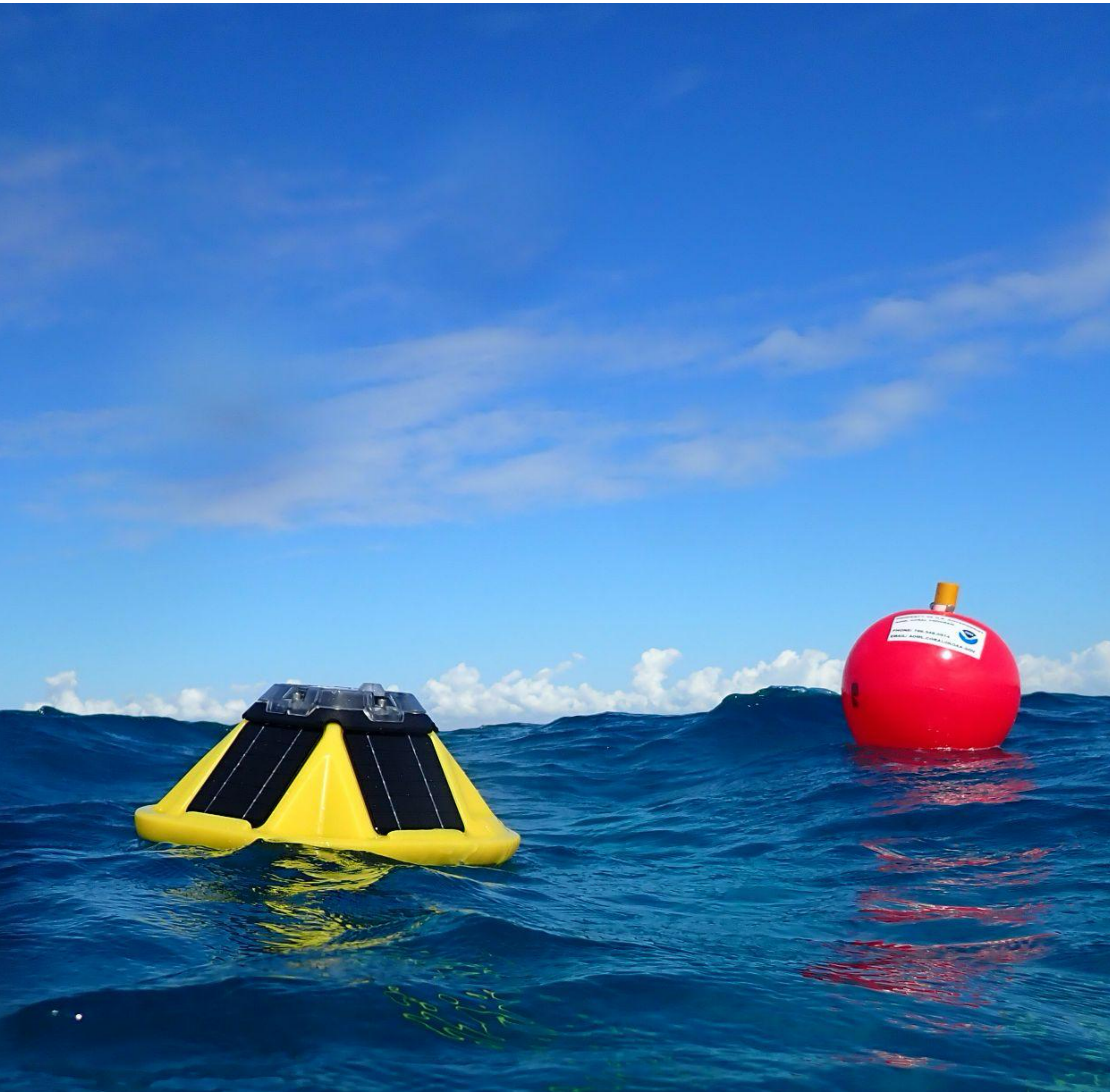

Build resources 3
System components 4
Bill of materials (BOM) 4
Buoy construction 5
Sofar buoy 5
SAMI-pH 11
DevKit wiring 11
Cable construction 13
Moorings 13
MAUI 13
Mooring pin 15
Software instructions 16
Firmware 16
API code 16
Shiny application 17
Deployment instructions 18
System testing 18
Mooring deployment 20
System connection 20
System deployment 20

Build resources

This system consists of a Sofar Spotter buoy (Figure S1a) paired with the Bristlemouth Development Kit (Figure S1c), both of which are available through Sofar Oceans. Detailed startup and assembly instructions for these components can be found in their official documentation. Additionally, the SAMI-pH sensor (Figure S1b) was sourced from Sunburst Sensors, LLC and installed following the guidelines provided in its manual.

This document provides a summary of our setup process along with additional insights into how we assembled and deployed the system.

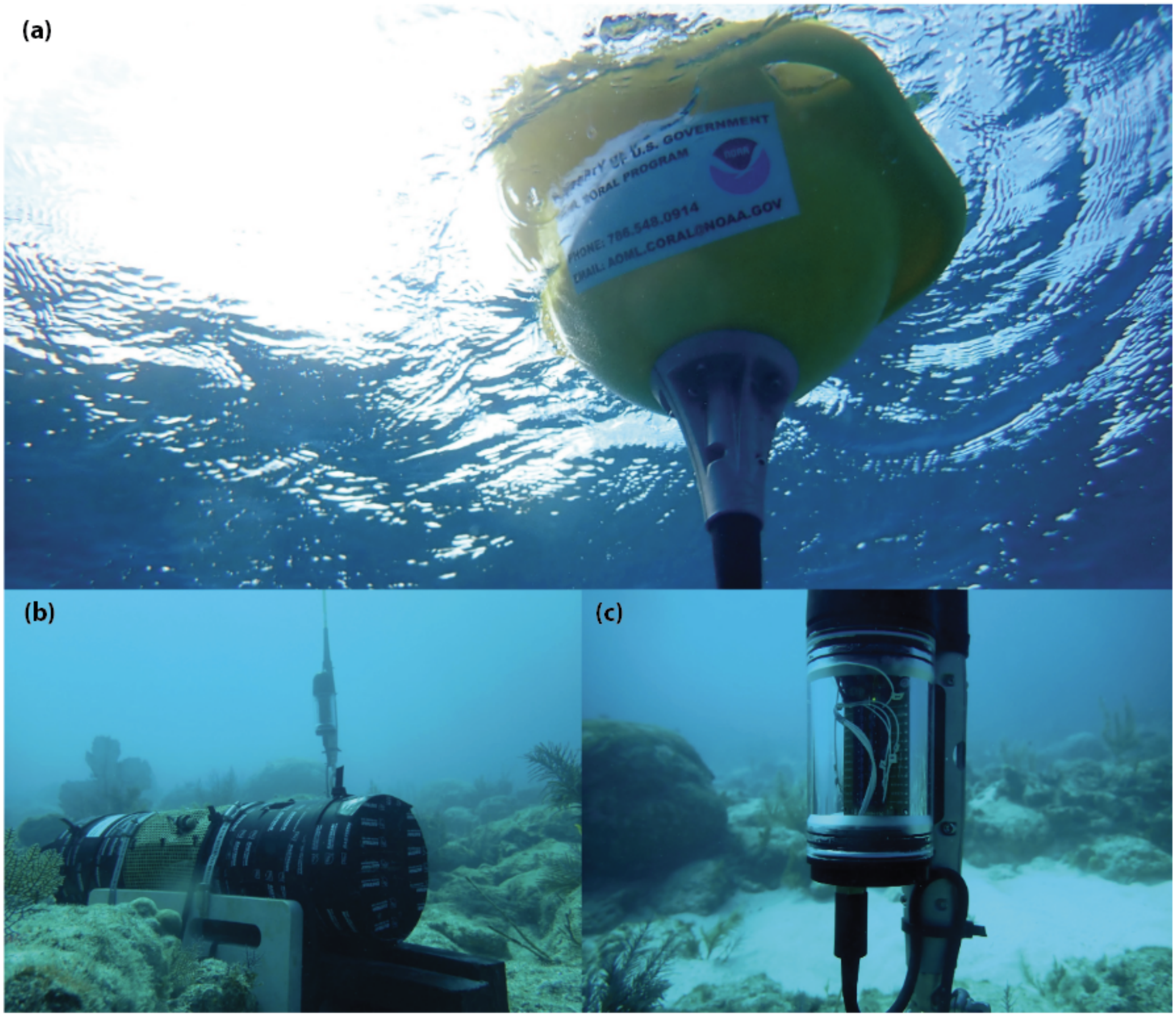

Figure S1. (a) Sofar Spotter buoy, (b) SAMI-pH sensor, and (c) Bristlemouth DevKit deployed at Carysfort Reef.

System components

This monitoring system has three primary components: a Sofar Spotter buoy designed for environmental data collection and cellular connectivity, a smart mooring cable that facilitates sensor communication, and a secure mooring platform for stable and consistent in-situ measurements. Each of these components play a critical role in enabling real-time, high-quality environmental data transmission.

1. Surface buoy system

- Sofar Spotter buoy - Records wave, wind, and barometric pressure data while transmitting information from additional instrumentation attached to the smart mooring downline.
- Temperature probe - Measures surface temperature and is integrated between the two mooring line segments.
- In-line float - Provides buoyancy for the DevKit.

2. Data transmission system

- Smart mooring cable - An underwater data cable that transmits sensor data to the buoy. Different cable lengths are available; for our ~20 ft deployment, we use five m and 10 m mooring lines.
- Custom communication cable - Connects the SAMI-pH sensor to the Devkit for data transmission.

3. Sensor platform and mooring system

- Bristlemouth DevKit - A modular interface for connecting and integrating non-Bristlemouth enabled sensors.
- SAMI-pH sensor - Measures seawater pH and benthic temperature.
- MAUI weights and mooring pin - Secures the underwater instrumentation and provides stability. For our deployment areas with variable wave height and weather events, our buoy is attached to the mooring pin, while the SAMI-pH sensor is moored solely by the MAUI weights.

Bill of materials (BOM)

**Component**	**Number**	**Cost per unit - USD**	**Total cost - USD**	**Source of materials**	**Material type**
Sofar Spotter Buoy	1	$6,600	$6,600	Sofar Oceans	Other
DevKit	1	$1,995	$1,995	Sofar Oceans	Other
Temperature Node	1	$795-$845	$795-$845	Sofar Oceans	Other

5 m Mooring Line	1	$550	$550	Sofar Oceans	Other
10 m Mooring Line	1	$650	$650	Sofar Oceans	Other
In-Line Float	1	$795	$795	Sofar Oceans	Other
Surface/Protection Float	1	N/A	N/A	Optional Addition	Other
SAMI-pH Sensor	1	$18,000	$18,000	Sunburst Sensors	Other
Communication Cable with Bulkhead	1	$1,129	$1,129	Teledyne	Belden
MAUI Mooring Weights	2	$649	$1,299	Local Welding Company	Lead
MAUI Mooring Side Mounts	2	$68.85	$68.85	USA Plastics	Starboard
MAUI Mooring Connectors: 1 bolt, 1 locking nut, 1 hex nut, 2 washers per point	4	$3.02	$14.96	Grainger	316T Stainless Steel
Mooring Pin	1	$6	$6	Local Welding Company	316T Stainless Steel

Table 1. Bill of materials (BOM) for the monitoring system.

The system supports two deployment configurations:

1. One stainless steel mooring pin with one MAUI Mooring set (the setup used in our deployments).
2. Two MAUI Mooring sets without a pin.

Buoy construction

Sofar buoy

This system design is easy to assemble and allows for rapid field deployments. This section will outline the step-by-step process to build the system, ensure all components are properly connected, and make sure all electrical pieces are protected and waterproofed.

1. Unboxing and preparation

- The Sofar Spotter buoy system comes preconfigured with all major components included.
- The kit includes two hex keys, required for securing/modifying each section.

2. Component overview

The system consists of the following components from top to bottom.

1. Spotter buoy
2. five m smart mooring cable
3. Center node housing the temperature probe
4. 10 m smart mooring cable
5. Bottom node

The DevKit, in-line float, and a surface protection float will be provided in separate boxes. These pieces can be seen below:

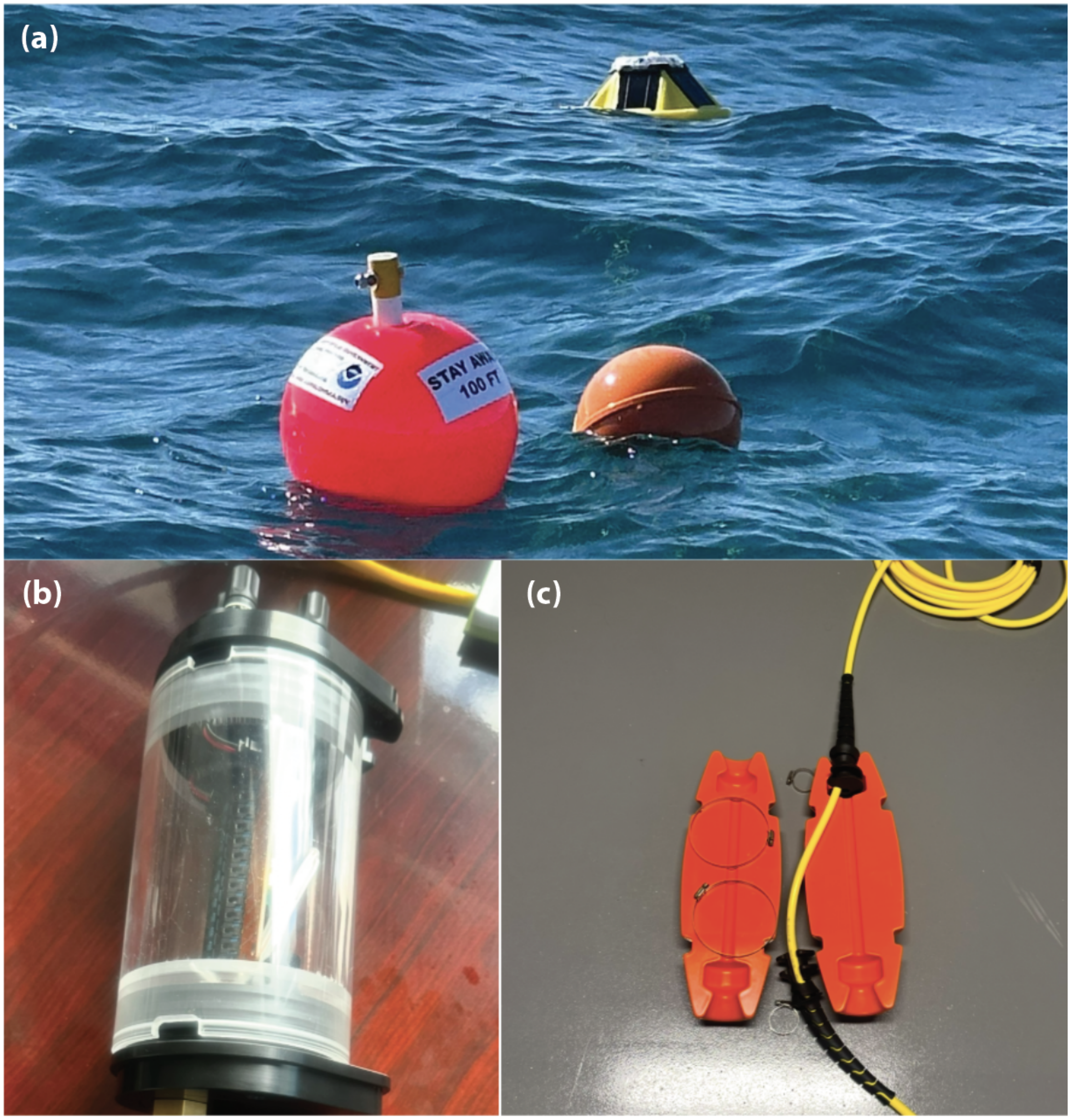

Figure S2: (a) The Sofar Spotter buoy deployed at sea, accompanied by two variations of a protection buoy. (b) The DevKit, which houses essential electronics and is secured to the bottom node during deployment. (c) The in-line float, prepared for attachment to the downline approximately one m above the bottom node, providing additional buoyancy and maintaining proper cable positioning.

Development Kit

The DevKit enables the integration of external instrumentation with the Sofar buoy by connecting directly to the smart mooring line. It interfaces with the data cable and provides a flexible platform for users to create additional connection points for instruments that are not equipped with Bristlemouth connectors. The kit houses a circuit board that can be custom wired to support specialized communication between instrumentation and the buoy system.

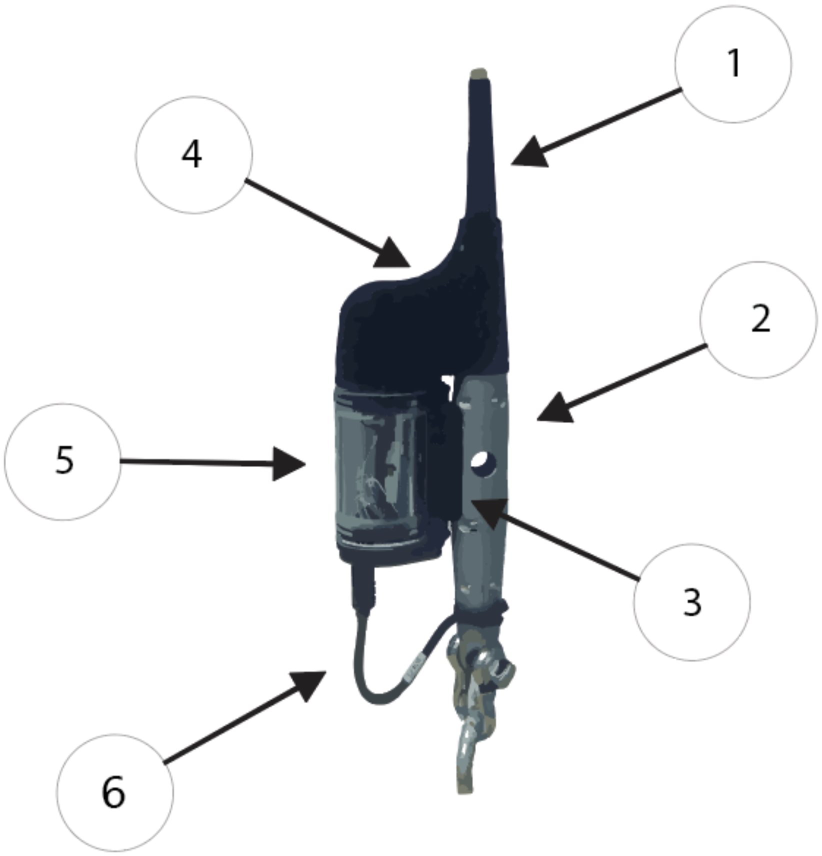

Figure S3. Annotated diagram of the DevKit. (1) Bottom end of the mooring line and data cable; (2) metal bottom node used to secure and support components; (3) mounting bracket for attaching the kit to the mooring infrastructure; (4) protective plastic cap/cover for the DevKit; (5) acrylic housing that contains and protects the circuit board; (6) custom communication cable connection point for Sofar/SAMI interface.

1. Component overview

The DevKit consists of the following components from top to bottom, numbered as specified in Figure S3.

1. End of 10 m smart mooring line
2. Metal bottom node
3. Mounting bracket
4. Cap/Cover
5. Acrylic circuit board housing
6. Custom communication cable connection point

2. Assembly and connection

The system arrives with pre-connected components, minimizing the need for extensive field setup. Jumper cables are already installed within the protective housings of the center and bottom nodes, linking each section of the smart mooring: from the Spotter buoy to the five m smart mooring cable, from the five m cable to the center node containing the temperature probe, then to the 10 m smart mooring cable, and finally to the bottom node.

To complete the assembly, the 300 mm jumper cable provided in the DevKit box is connected to the end of the 10 m cable (Figure S3.1). This jumper is threaded through the oval-shaped hole in the bottom node (Figure S4a) and securely attached to the top of the DevKit (Figure S4b).

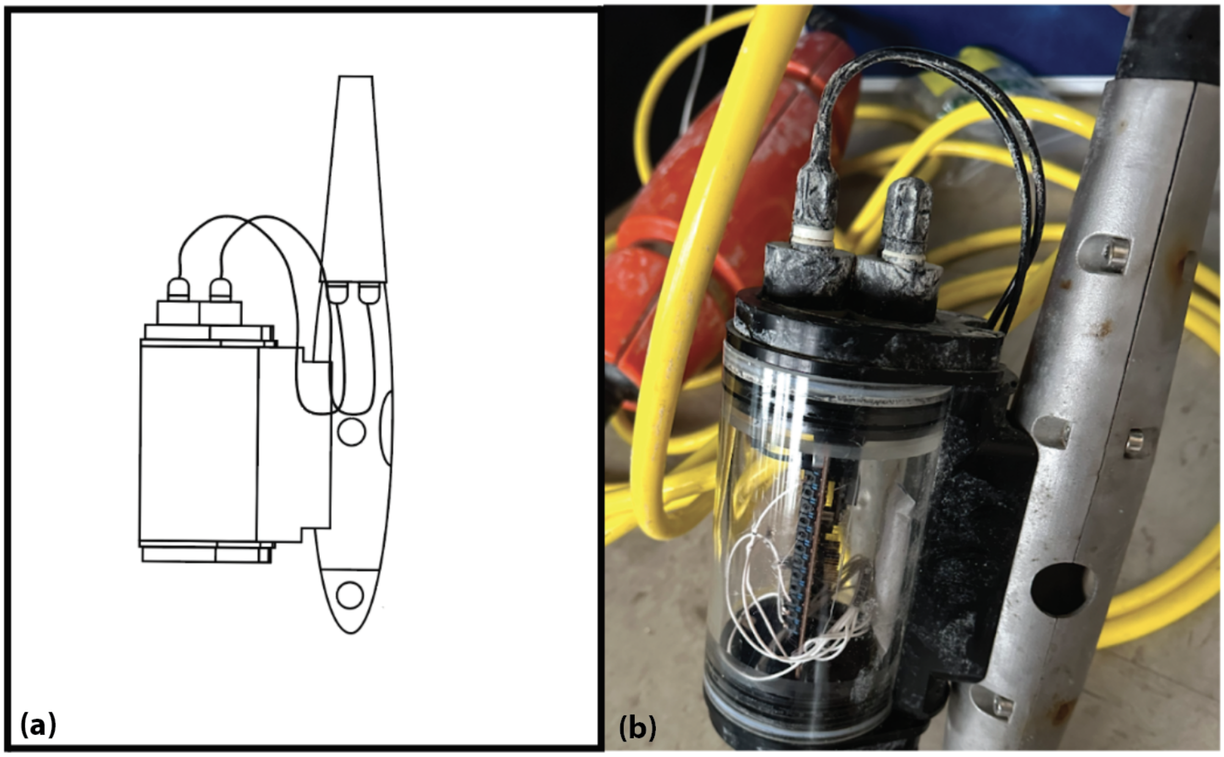

Figure S4. (a) Diagram of 300 mm jumper cable wiring. (b) 300mm cables securely attached to the top of the DevKit. These cables are threaded behind the kit into the bottom metal node, where the other end connects to the 10 m downline, ensuring proper electrical and mechanical integration.

3. DevKit installation

The DevKit is secured to the bottom node using a black mounting bracket (Figure S3.3). This bracket is installed onto the node with the provided hex key, allowing for precise positioning and secure fastening. A black plastic cap (Figure S3.4) is fitted onto the top of the DevKit, wrapping around the mooring line to protect the jumper cables from external elements. Once secured, the cap is fastened using zip ties to prevent movement during deployment.

Protection float installation

The protection float is an optional component of the moored system that attaches to the center node of the Sofar buoy (Figure S5a) using a swivel shackle. Both the float and shackle are supplied by Sofar Ocean. Depending on site-specific environmental conditions, a custom float or alternative shackle may be used.

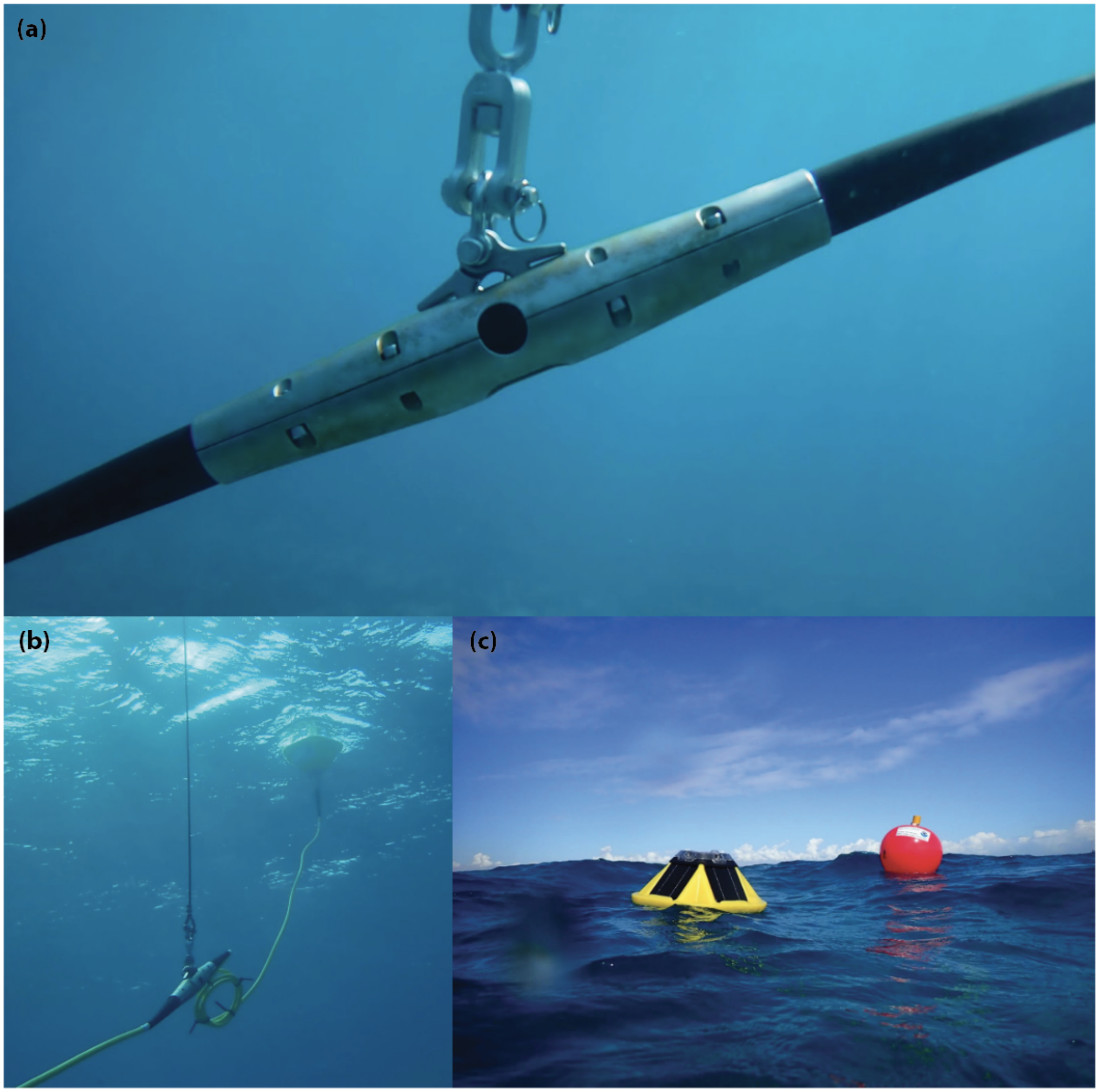

Figure S5. (a) Swivel shackle attached to the center node. (b) The protection buoy is attached to the center metal node, providing buoyancy and stability to the mooring system. (c) The Sofar Spotter buoy and the protection buoy floating at the surface.

Modification for SAMI-pH sensor integration

To integrate the SAMI-pH sensor, a hole was drilled into the DevKit bottom cap made of polyoxymethylene, commonly known as acetal, to accommodate a bulkhead fitting (Figure S6b). The hole was positioned near the outer edge of the indented safe-drilling area on the rounded side of the cap to allow the bulkhead cables to route cleanly towards their connection points on the circuit board. Exact placement is not critical provided the fitting does not interfere with internal hardware. A 3/16-inch drill bit was first used to create a pilot hole, which was then expanded with a 25/64-inch bit to the required size. A 7/16-20 tap was used to cut internal threads for securing the bulkhead fitting. Teflon tape was applied to the threads before installation to ensure a watertight seal. Additional details on this process are available in the Bristlemouth documentation.

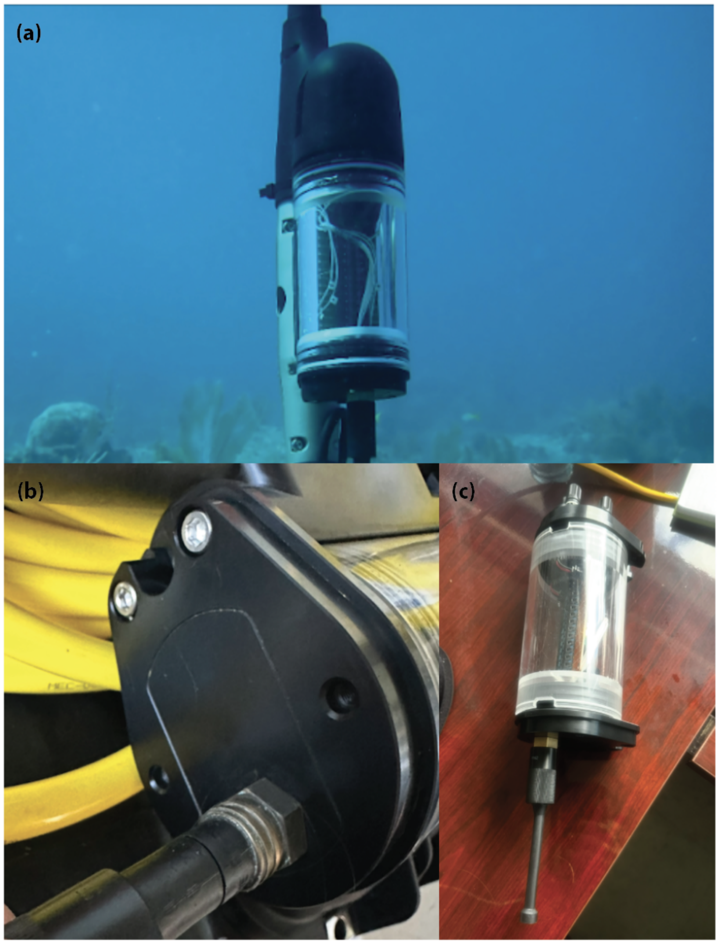

Figure S6. DevKit integration and deployment. (a) DevKit mounted to the metal bottom node and deployed underwater. (b) Close-up of bulkhead fitting screwed into the base of the DevKit for sensor integration. (c) DevKit, showing the bulkhead fitting with a communication cable dummy plug inserted for sealing during non-operational periods.

Waterproofing and pressure testing

Before deployment, the modified DevKit underwent pressure testing to verify its waterproofing. The unit was pressure-tested in-house at 2.5 Bar (36 PSI), equivalent to a depth of ~25 m, for three days. Prior to testing, the circuit board was removed, and all electrical wires were sealed with electrical tape for added protection. After testing, the system was inspected for signs of water ingress. If no leaks were detected, the kit was reassembled and prepared for field deployment.

SAMI-pH

Before deployment, the SAMI-pH sensor is wrapped with PVC anti-biofouling tape to prevent biological growth. Avoid covering the copper cage to maintain proper water flow and accurate measurements. Additionally, eight cutouts are made on the bottom face of the SAMI-pH (Figure S7a) and four are made below the copper cage around the sides of the SAMI (Figure S7b) to avoid air bubble formation and decrease the chance of the instrument becoming too positively buoyant. In various field deployments, this anti-biofouling tape has remained intact for multi-year periods without requiring replacement; its primary function is to facilitate post-recovery cleaning and provide general housing protection rather than to maintain intake flow performance.

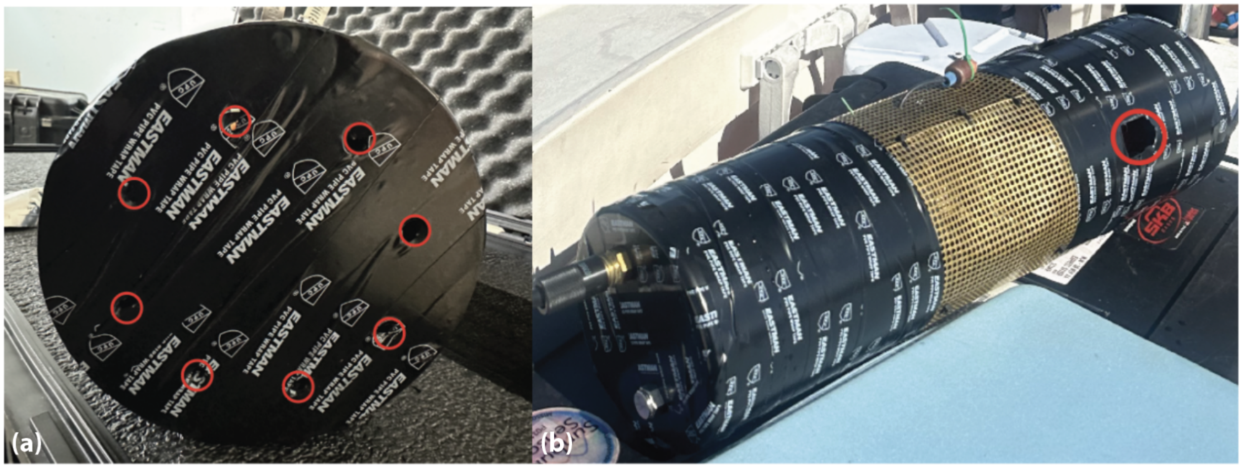

Figure S7. SAMI prepped for deployment and wrapped in antibiofouling tape with (a) holes cut out in the bottom and (b) sides.

DevKit wiring

After passing the pressure test, the circuit board within the DevKit was wired to establish an RS-232 communication configuration between the SAMI-pH sensor and the DevKit. This configuration ensures reliable data transfer between the instruments.

Inside the DevKit, the following wiring connections were made:

Pin 1 → Ground
Pin 2 → Tx (Transmit)

Pin 3 → Rx (Receive)
Pin 6 → Data Ground

The RS-232 pinout on the SAMI-pH follows this communication scheme and integrates with the DevKit. It is essential to verify that the Tx output from the SAMI-pH is connected to the Rx input on the circuit board, and vice versa, to establish proper signal transmission.

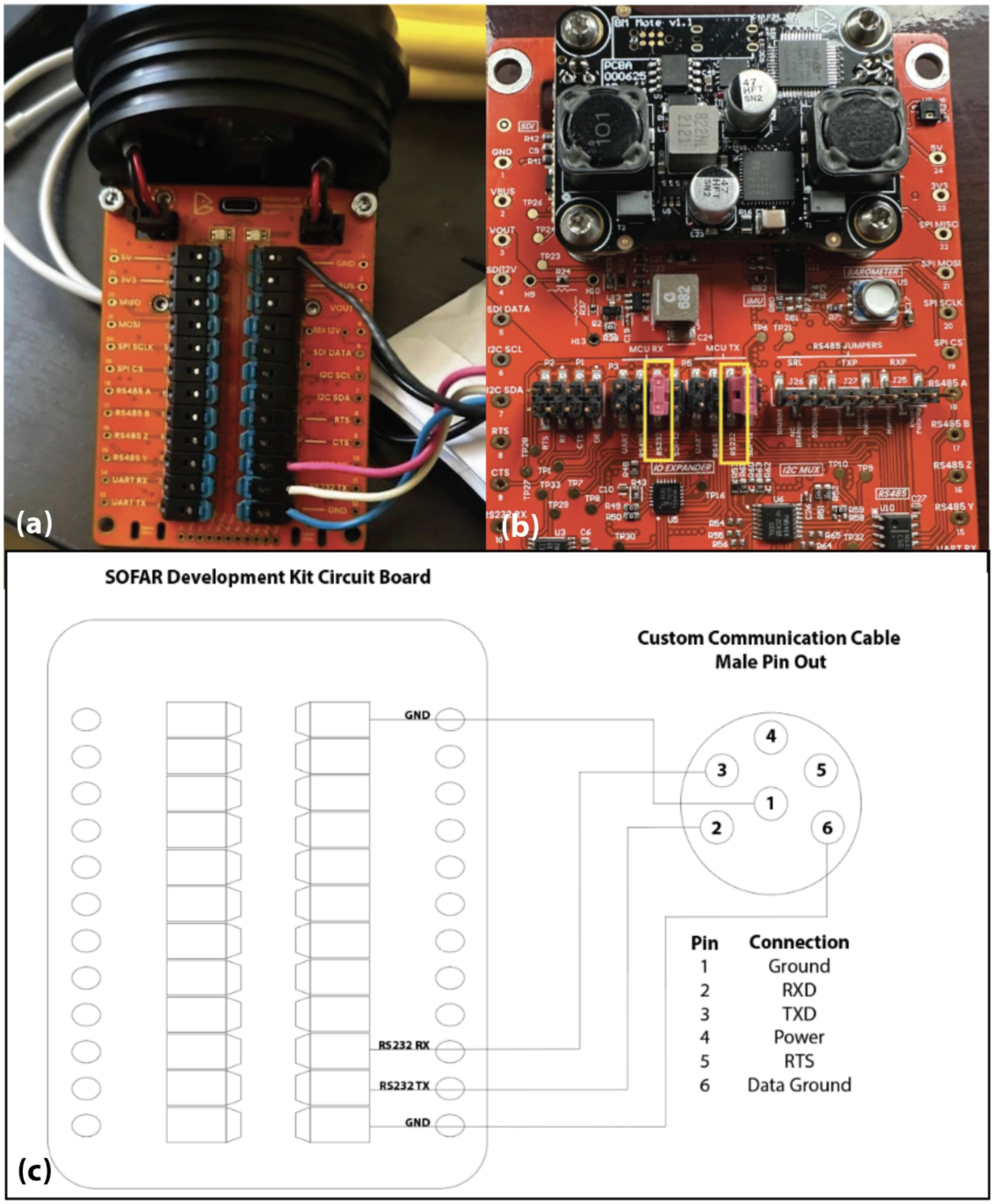

Figure S8. (a) DevKit with custom SAMI-pH cable wiring in RS-232 communication scheme. (b) Back of DevKit circuit board with jumper connections. (c) Diagram of SAMI-pH pin out and connection to circuit board in RS-232.

Once the wiring is done, the DevKit was sealed with O-rings and lubricant as well as the provided locking rings. Once fully sealed it was screwed into place on the mount on the bottom node, covered with the cap and connected to the custom communication cable.

Cable construction

The custom communication cable was designed to provide a secure, durable, and interference-free connection between the SAMI-pH sensor and the DevKit. This cable was assembled by molding two micro in-line six-pin male impulse connectors (MCIL-6-MP), with one m long pigtails, on either side of two m of additional cable (Figure S9a). The total cable length is ~three m, ensuring flexibility while preventing excess slack that could lead to entanglement.

The cable is constructed using Belden marine-grade wiring. To enhance durability and protect against abrasion from the seafloor and other environmental factors, the entire length of the cable is wrapped in a high-strength spiral sleeve made from high-density polyethylene (HDPE) plastic (Figure S9b). This outer covering provides resistance to physical wear while maintaining flexibility for deployment in dynamic ocean conditions.

Both ends of the cable are equipped with micro wet-connection Delrin female locking sleeves, ensuring secure and waterproof connections to the SAMI-pH and the DevKit bulkheads. The locking mechanism prevents accidental disconnections, even under strong currents or turbulent conditions. Additionally, a six-pin female bulkhead was included in the setup, replicating the SAMI-pH bulkhead with a six-wire flow-through configuration that allows direct wiring to the DevKit circuit board.

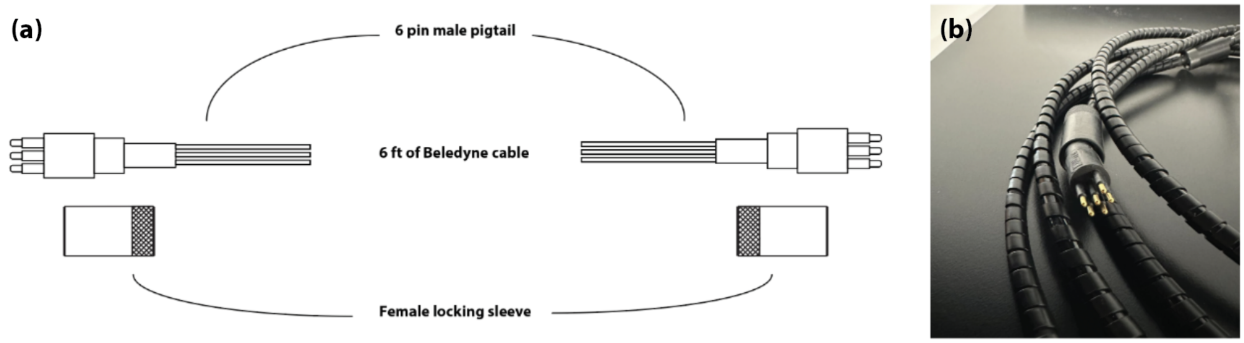

Figure S9. (a) Diagram of custom cable creation. (b) Image of cable ready for deployment with high-density polyethylene (HDPE) protective plastic on top and six-pin connection showing.

Moorings

MAUI

The MAUI weight system was designed by the NOAA Fisheries Hawaii team as a solution for securely mooring marine instrumentation in a streamlined fashion. This setup consists of two lead weights, each weighing 70 lb, for a combined total of 140 lb. The weights are coated with

marine paint to resist corrosion and biofouling, ensuring long-term durability. Each weight is equipped with ¾-inch thick starboard side mounts that are secured with stainless steel bolts, hex nuts, locking nuts, and washers. This design is adaptable to various marine environments, allowing the buoy and SAMI-pH to be anchored safely.

The SAMI-pH is secured to the MAUI weight system with two hose clamps tightened to the starboard side mounts and four zip ties secured around the weights. The buoy bottom node is connected to the MAUI weight system with a stainless steel chain. The chain is threaded through the weight system and connected to a stainless steel D-shackle. Two additional wire rope clips are added to the ends of the chain to ensure underwater security and system connection.

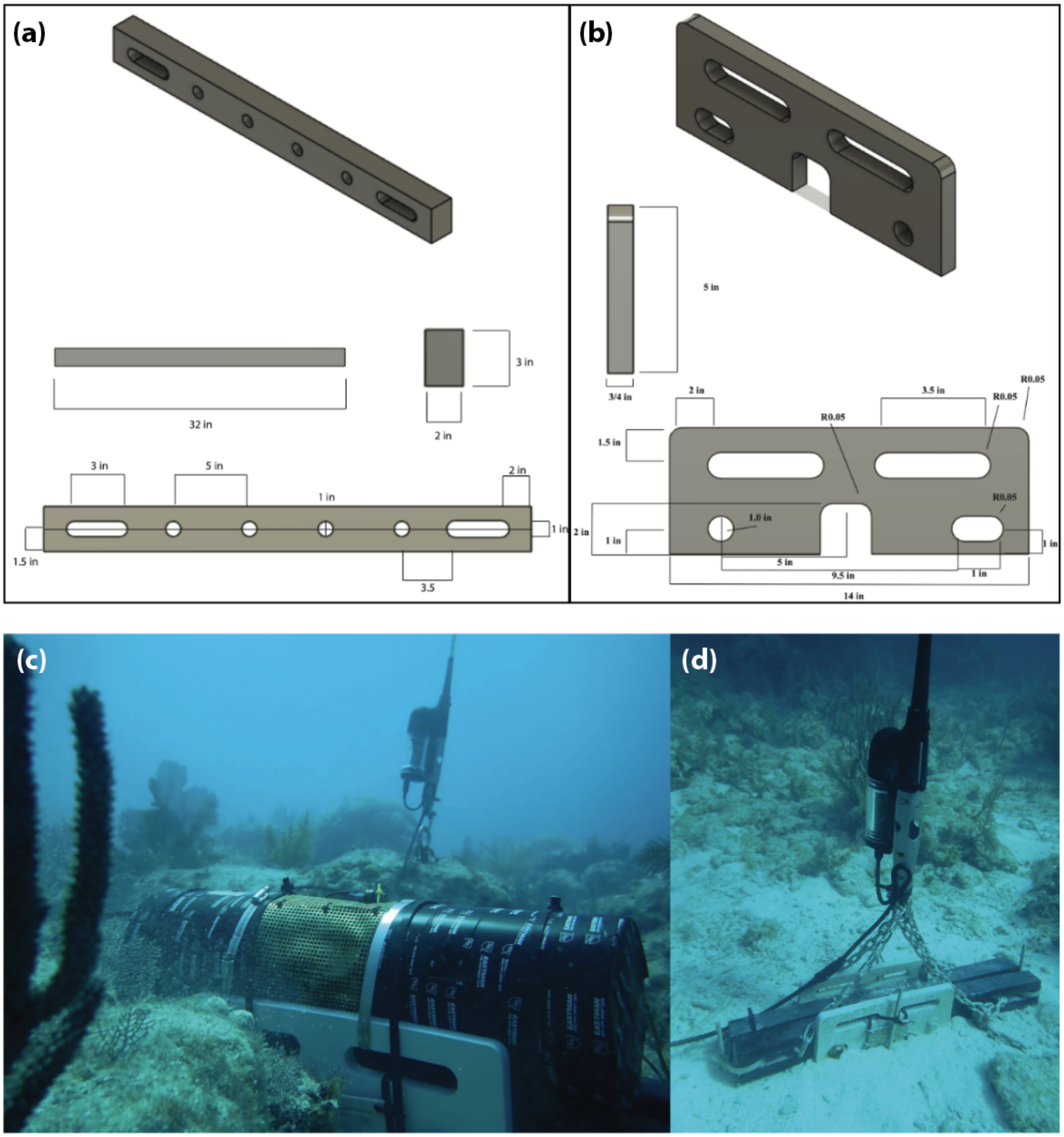

Figure S10. (a) Schematic of 70 lb lead weights and (b) starboard side mount that go together to create the MAUI weight system. (c) SAMI-pH and (d) buoy moored on MAUI weights.

Mooring pin

Alternatively, a 5/8-inch thick 21 1⁄2- inch long stainless steel mooring pin with a T-fitting on the eye is used in certain deployments to provide additional anchoring support in areas with strong currents or wave activity. The pin is drilled into the substrate through the use of a NEMO Hammer Drill and a SDS chuck adapter. The hole is drilled by a two-inch core bit to fit the pin. The hole is then filled with a cement mixture using Type I/ Type II Portland cement with a one-to-one ratio until a sticky, but not runny consistency is achieved. This is brought down in 16-inch piping bags to provide clean deployment and installation. After letting the cement harden for 48 hours, the buoy is then connected to the mooring system via its bottom node using a stainless steel D-shackle. D-shackles are preferred over swivels to minimize torque on the communication cable, which links the buoy to the SAMI-pH. To prevent corrosion, all connection points are made with matching 316L stainless steel components, enhancing the longevity and reliability of the system. Additionally, to prevent overextending the custom communication cable, it is zip tied to the MAUI weights and buoy bottom node to provide extra slack so that it is never fully extended at vital connection points. Lastly, the stainless steel D-shackle is locked in place with a zip tie to prevent any disconnection under water.

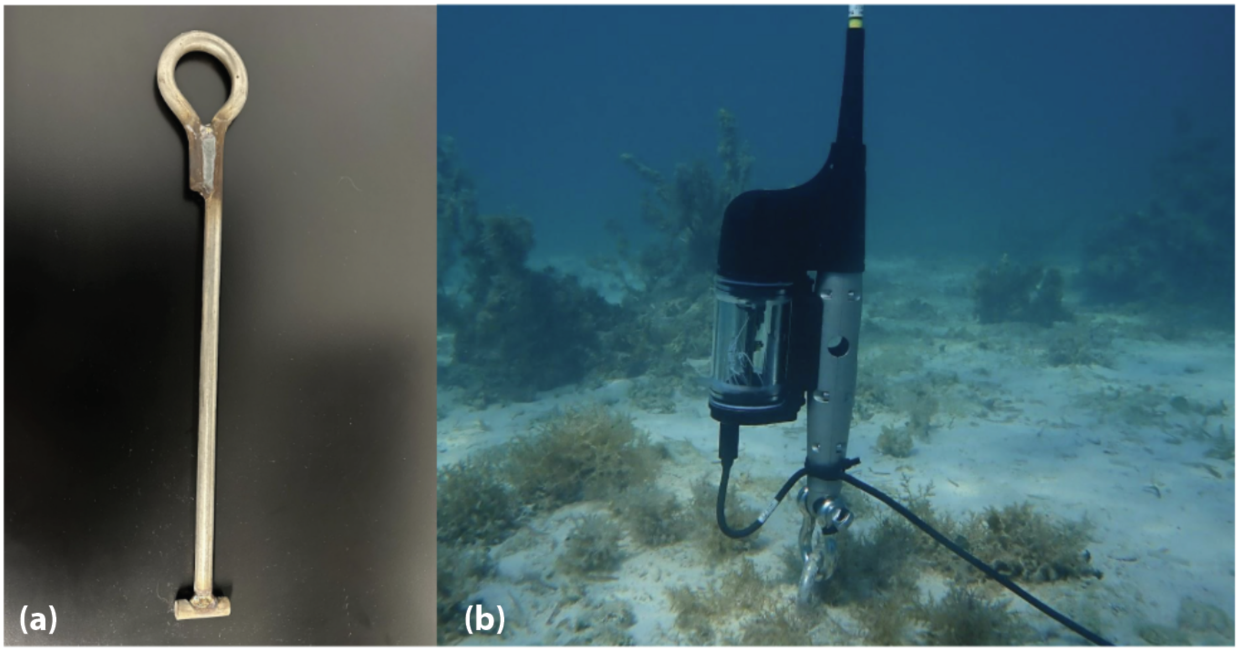

Figure S11. (a) Stainless steel mooring pin. (b) Buoy bottom node deployed on mooring pin.

Software instructions

Firmware

This system uses the C++ source code for the DevKit firmware, and the python script that is used to connect to the Sofar API, download the data, and process the raw SAMI-pH strings into values.

For the C++ source code, Bristlemouth provides extensive guides and examples on their website about how to customize your own firmware to integrate sensors, compile your code into binary firmware, and flash the resulting firmware to the Bristlemouth mote in the DevKit. Our code borrows heavily from their 'hello world' and 'serial payload' examples, but reads serial input character by character rather than one line at a time. Coding and testing the firmware involves connecting the full integration (SAMI-pH, custom communication cable, and DevKit) and configuring it via a command-line interface (CLI) through the use of a USB-C data cable. The firmware binary file customized for Sofar-SAMI communication is loaded onto the SD card of the spotter buoy and then flashed to the Bristlemouth mote on the DevKit to update the entire system. Use the following code in the CLI to finalize the firmware update:

bridge dfu (Custom firmware code) 0x(DevKit node ID) 120000 force

Additionally, we set a specific configuration of the Sofar Spotter buoy to carefully align sampling with the SAMI-pH settings. Enter the following code into CLI to configure the buoy to turn on every hour and sample for 10 minutes:

bm cfg set 0 s u bridgePowerControllerEnabled 1
bm cfg set 0 s u sampleIntervalMs 3600000
bm cfg set 0 s u sampleDurationMs 600000
bm cfg set 0 s u samplesPerReport 1
bm cfg commit 0 s

This setup ensures that the Spotter buoy's sampling schedule matches the SAMI-pH's hourly measurements, enabling synchronized data collection. The configuration also optimizes power use by enabling the bridge power controller and limiting sampling duration to conserve energy and extend deployment longevity. These detailed programming steps ensure reliable communication and data collection between the instruments during extended deployments.

API code

Our sample API code is an example of how to connect to the Sofar API on a regular basis and download new data from your deployed buoys. The sample code must be configured with the identifying information for your equipment. At the top of the file are these lines of code:

# hardcoded parameters for demonstration purposes

site_name = 'xxxxxxxxx'
spotter_id = 'SPOT-xxxxxC'
api_token = 'xxxxxxxxxxxxxxxxxxxxxxxxxxxxxxxxx'
config_info = 'xxxxx […] xxxxx'

The site_name is a descriptive string to help distinguish between multiple buoy sites. The spotter_id and api_token are specific to each buoy and may be obtained from the Sofar online dashboard. The config_info is a hexadecimal string that is written into every SAMI-pH configuration file during sensor setup. It is 104 characters and appears following the line ':SAMIinfoHex' near the bottom of the file. Note that this string is SAMI-specific and must be kept current in your software configuration whenever a SAMI is deployed or swapped.

This code example by default looks for the past six hours of data, and it writes the different types of data (wind, waves, barometric pressure, surface temperature and SAMI-pH parameters) to different files in CSV format. In our system this code forms a part of a larger infrastructure involving a MySQL database and a front-end dashboard coded in R/Shiny. The database tracks all the buoys and their locations, their spotter IDs and API tokens, and the serial numbers and config_info strings of their associated SAMIs over time. The python code can therefore check the database history to determine when the last data updates of each data type occurred, and adjust the Sofar API URL's query parameters to ask only for data reports that are newer than data already seen. Our python code is scheduled to check for new incoming data every five minutes.

Shiny application

The data transmitted from the Spotter buoys, over cellular, is then publicly displayed through an interactive dashboard designed using R Studio and the Shiny framework (Figure S12). The Shiny app connects directly to a MySQL database, retrieving and displaying the latest data as it becomes available.

The dashboard features multiple pages, offering a comprehensive view of the monitored reefs. One page displays all reefs on the same interactive plots, allowing users to compare data across locations, with each reef distinguished by unique colors. Additional pages provide site-specific information, including individual plots of pH, temperature, wave height, and other parameters for a selected reef.

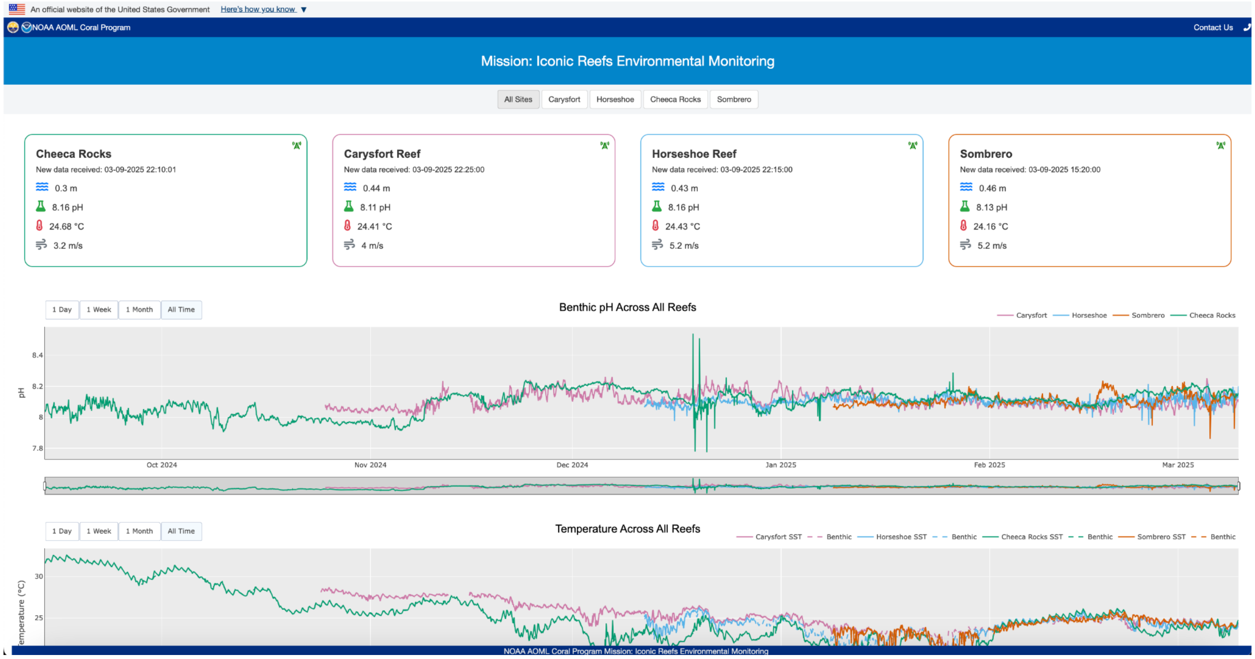

Figure S12. Public dashboard displaying data coming from each of the Mission: Iconic Reef Environmental Monitoring buoys. This can be accessed at: https://coral.aoml.noaa.gov/mir/. Data depicted in this figure are preliminary real-time measurements and have not undergone full post-deployment calibration or quality control.

Deployment instructions

Safety: Personnel should be aware of the following hazards: (1) electrical hazards associated with operating an electronic system in a marine environment; (2) chemical exposure during methanol handling for filter flushing; (3) irritation from cement mixtures; (4) use of underwater power tools; and (5) marine environment hazards during SCUBA deployment. Appropriate PPE and safety protocols should be followed.

System testing

The SAMI-pH undergoes in-house calibration and preparation. Calibration includes a two-day process in which the SAMI is placed in a water bath with constant water circulation provided by a water pump. The SAMI acclimates during the first 24 hours and on the second day, three bottle samples are collected at two-hour intervals. These samples are analyzed in the laboratory for pH and salinity through spectrophotometry and densitometry, with additional validation provided by subsurface temperature recorders (STR) and YSI measurements taken alongside the SAMI.

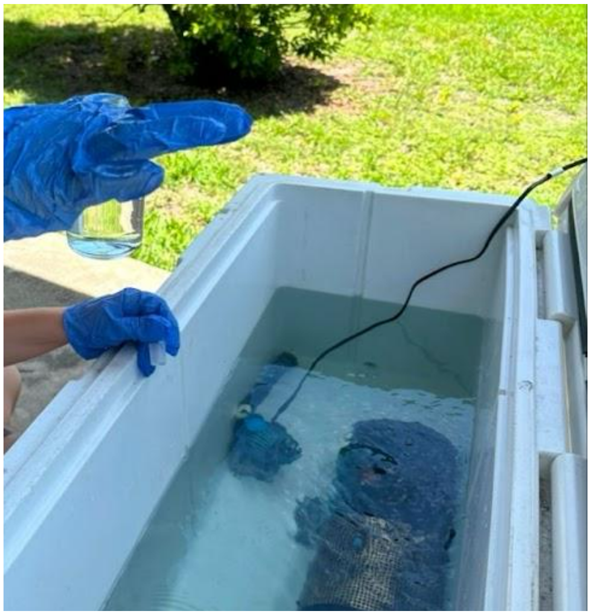

Figure S13. SAMI-pH going through an in-house calibration.

Once the whole system is connected it undergoes a 24 hour energy usage and data transmission test. Everything is set up and the SAMI sits in a salt water bath. Make sure the buoy is in direct sunlight with nothing obstructing it from the open sky. Let the System run for 24 hours. Make sure all data is coming in on time and check the energy levels to ensure it is recharging properly and can handle the data income load. If you follow our system design the battery should handle the data load.

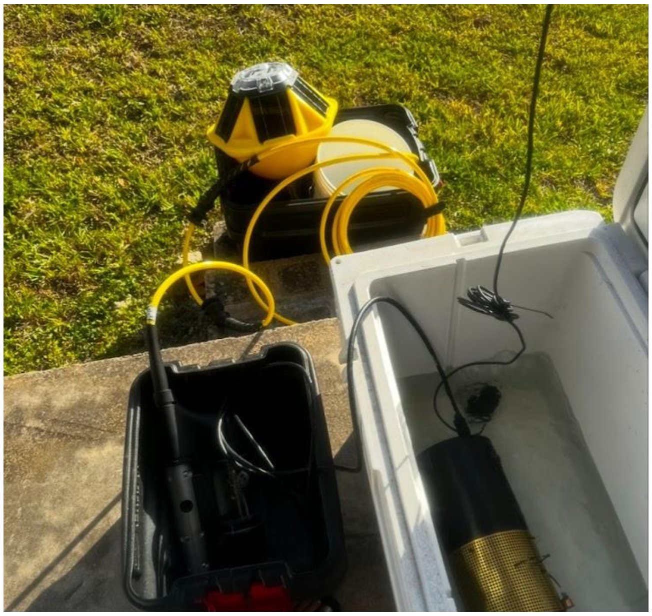

Figure S14. SAMI-pH integrated Sofar buoy undergoing an energy and data transmission test.

Check the day before deployment to ensure there is no air lock occurring in the SAMI-pH tubing. Connect the SAMI to a computer and open the SAMI Client Software. Under the utility tab set the number of cycles to 10 and connect a syringe full of DI water to the copper bell. Holding constant pressure hit run and let the SAMI pump the DI water through its system. If the water flowed through and there was no blockage then set the number of cycles to 99. For this one you can either put the bell in a cup of DI water or attach its bag and let it run through the system. Once the SAMI finishes, as long as there are no blockages it is ready for deployment. DI water and Methanol is kept safely in the SAMI case to flush the copper filter before deployment. On the day of deployment use the syringe to do three rinses of methanol followed by three rinses of DI through the filter before attaching it to the copper bell and securing it with a zip tie to the copper cage. This methodology can be found in more detail in the SAMI-pH manual. Without these flushes the filter will not be properly wet, and this could lead to the first few samples being inaccurate. Our team usually considers the first few hours of data to be inaccurate as the instrument acclimates.

Mooring deployment

Depending on the site of deployment, choose what type of mooring you are going to install. The first step in deployment will be installing the mooring. If it is the MAUI weights, make sure everything is set up and in place underwater before deploying the instruments. If using the pin, you will need to install it two days before instrument deployment to allow for the cement to harden.

System connection

Attach the buoy and SAMI to the communication cable in air. Have two divers in the water. Have one hold the SAMI-pH while being mindful of the filter, and one hold the bottom node with the DevKit. Have the team on the boat put the rest of the buoy in the water as the dive team brings the equipment underwater.

System deployment

One diver should attach the buoy bottom node to the designated underwater anchor point, whether that be a mooring pin or a MAUI weight setup. The node should be secured to a ½" D-shackle, and the bolt should be zip-tied or otherwise secured to prevent loosening over time. Simultaneously, a second diver should attach the SAMI-pH sensor. The SAMI should be positioned between the two side mounts and secured using two large hose clamps. For reference, a combination of 13/16-1¾ inch and 3⅛-6 inch hose clamps were successfully used by linking them together. The hose clamps should be looped through the side mount brackets and tightened to secure the SAMI in place. Additionally, two to four zip ties should be wrapped around the SAMI and the mooring weights for added stability. Zip ties should also be used to secure the ends of the cable to both the SAMI mooring and the buoy's bottom node to ensure the cable is never fully extended, which helps prevent stress on critical connection points.

After deployment, an underwater water sample should be collected near to the SAMI-pH at the moment it takes a reading (i.e., on the hour). This sample will be used for post-deployment validation of the instrument's measurements.
